# Diet Quality of Elite Australian Athletes Evaluated Using the Athlete Diet Index

**DOI:** 10.3390/nu13010126

**Published:** 2020-12-31

**Authors:** Louise Capling, Ryan Tam, Kathryn L. Beck, Gary J. Slater, Victoria M. Flood, Helen T. O’Connor, Janelle A. Gifford

**Affiliations:** 1 Faculty of Medicine and Health, Sydney School of Health Sciences, The University of Sydney, Sydney, NSW 2141, Australia; acap7726@uni.sydney.edu.au (L.C.); ryan.tam@sydney.edu.au (R.T.); vicki.flood@sydney.edu.au (V.M.F.); helen.oconnor@sydney.edu.au (H.T.O.); 2 Sport Performance Innovation and Knowledge Excellence Unit, Queensland Academy of Sport, Nathan, QLD 4111, Australia; 3 School of Sport Exercise and Nutrition, College of Health, Massey University, Auckland 0632, New Zealand; k.l.beck@massey.ac.nz; 4 School of Health and Sport Sciences, University of the Sunshine Coast, Sippy Downs, QLD 4556, Australia; gslater@usc.edu.au; 5 Charles Perkins Centre, The University of Sydney, Camperdown, NSW 2006, Australia; 6 Western Sydney Local Health District, Westmead, NSW 2145, Australia; 7 South Western Sydney Local Health District, Liverpool, NSW 2170, Australia

**Keywords:** dietary assessment, sports nutrition, athletes, exercise

## Abstract

While athletes’ nutrient intakes have been widely reported, few studies have assessed the diet quality of athletes. This is the first study to evaluate the diet quality of athletes using the purpose-built Athlete Diet Index (ADI). A convenience sample of 165 elite athletes from Australian sporting institutions completed the ADI online, with subsequent automated results provided to their respective accredited sports dietitians (ASDs). At the completion of athlete participation, ASDs (*n* = 12) responded to a range of survey items using a Likert scale (i.e., 1 = strongly agree to 5 = *strongly disagree*) to determine the suitability of the ADI in practice. Differences in ADI scores for demographics and sport-specific variables were investigated using independent *t*-tests, analysis of variance (ANOVA) and Bonferroni multiple comparisons. Spearman’s rank correlation was used to assess the association between total scores and demographics. The mean total ADI score was 91.4 ± 12.2 (range 53–117, out of a possible 125). While there was no difference in total scores based on demographics or sport-specific variables; team sport athletes scored higher than individual sport athletes (92.7 vs. 88.5, *p* < 0.05). Athletes training fewer hours (i.e., 0–11 h/week) scored higher on *Dietary Habits* sub-scores compared with athletes training more hours (≥12 h/week; *p* < 0.05), suggesting that athletes who train longer may be at risk of a compromised dietary pattern or less than optimal nutrition practices that support training. Most (75%) ASDs surveyed *strongly agreed* with the perceived utility of the ADI for screening athletes and identifying areas for nutrition support, confirming its suitability for use in practice.

## 1. Introduction

Sports dietitians play a key role in delivering personalised nutrition advice and education to athletes in their care [1,2,3]. Dietary assessment is an important element of sports nutrition practice, helping to identify athletes at risk of nutritional inadequacy (i.e., athletes following restrictive diets and/or with low energy intakes), and to guide nutrition interventions. However, detailed dietary assessment methods, such as the food record (FR), dietary recall and in-depth interviews, are time consuming and difficult to undertake accurately in athletes [4,5,6]. Additionally, the provision of nutrition servicing in high-performance sporting programs is often limited by funding and the allocation of resources [1,2,7]. Technology-based dietary assessment methods have the potential to reduce the time associated with dietary assessment [8], enhance participant compliance [6,9], and allow real-time data collection [10], thereby offering a practical and efficient approach for sports dietitians who typically support numerous athletes in high-performance sporting programs.

To date, there has been limited evaluation of the adequacy of dietary intake [11,12,13], or dietary patterns [13,14,15,16] of athletes. Most dietary intake studies involving athletes have focused on achieving the prescribed guidance for energy and macronutrients compared to sports nutrition recommendations [17,18,19,20,21], or population nutrient reference values [19]. However, quantitative nutrient data may provide limited information regarding overall nutritional adequacy, particularly in athletes with very high or low energy intakes [19]. More recently, the application of diet quality indices such as the *Healthy Eating Index* (HEI) [22,23,24,25,26,27], and the *Australian Recommended Food Score* (ARFS) [12,28] have been used to evaluate the diet quality of athletes. However, these population indices typically assess dietary intake compared to population guidelines, and do not consider the specific dietary requirements (such as higher energy and nutrient requirements) or dietary habits of athletes engaged in a vigorous training program. While athletes are encouraged to follow a dietary pattern that meets population guidelines for general health and well-being [3,29]; athletes are also required to meet the energy demands of training, including the optimal timing of key macronutrients, such as carbohydrate and protein, to support training across the day [3,30]. Some athletes, for example adolescent and female athletes, may also have an increased need for micronutrients such as calcium and iron [3,29,31]. Furthermore, in recent years, the negative impact of low energy availability on health and performance of athletes has been widely documented [32,33,34], and is an important consideration when undertaking dietary assessment in athletes.

The Athlete Diet Index (ADI) is a valid and reliable diet quality assessment tool [35] that was developed specifically for elite athletes [36]. It rapidly evaluates athletes’ usual dietary intake, compares responses to population [37] and sports nutrition recommendations [3], and provides athletes with real-time feedback. Additionally, dietary components that reflect the intake of nutrients relevant to athletes (i.e., calcium, iron), and an evaluation of dietary habits that support a regular training program, are assessed and then summed to provide an overall score. The ADI automatically generates a detailed summary of the athletes’ overall results, in addition to capturing other non-scored information, for further interpretation by their sports dietitian. The aim of this study is to assess the diet quality of elite Australian athletes for the first time using the recently validated ADI, and to evaluate the perceived usefulness of the electronic tool by sports dietitians and the suitability for its use in sports nutrition practice.

## 2. Materials and Methods

### 2.1. Study Design

This cross-sectional study involved the delivery of a suite of nutrition assessment tools via an online platform (i.e., Accelerated Sports Nutrition Assessment Platform; ASNAP), comprised of three components: (1) demographics and sport-specific characteristics; (2) a sports nutrition knowledge tool (i.e., Platform to Evaluate Athlete Knowledge of Sports Nutrition Questionnaire, PEAKS-NQ) [38]; and (3) the ADI [36]. Athlete participants completed all three ASNAP components; however, data collected from the PEAKS-NQ will be reported elsewhere. The online platform was developed using FileMaker^TM^ Pro 16 software program (FileMaker, Inc., 2017, Santa Clara, CA, USA) and was administered via a web link housed on a university server. This study was approved by the University of Sydney Human Research Ethics Committee (protocol number: 2019/800). All participants provided written informed consent prior to their participation in this study.

### 2.2. Participants

A formal invitation seeking athlete participation was initially sent via e-mail to a convenience sample of accredited sports dietitians (ASDs) of sporting programs at Australian state-based sporting institutions. The ASDs who consented to participate in this study agreed to use the ADI with athletes in their sporting programs. Elite athletes (≥16 years of age) who had competed at state level or higher in their chosen sport were eligible to participate in this study. The athletes who consented to participate were subsequently provided with a unique participant ID generated by their nominated ASD and were provided with the log in details to access the online dietary assessment tools. Athletes completed the online ASNAP components at one time point. An initial reminder was sent via e-mail to the athletes with incomplete data within three days of their last online entry, with a second reminder sent seven days following their last online entry. Records that were not completed within 14 days were excluded from the analysis. Upon completion of the ADI, athletes automatically received a brief summary of their results via e-mail. If the athlete provided prior consent, a copy of their results was also sent to their nominated ASD via e-mail. 

### 2.3. Assessment of Diet Quality

The ADI scoring matrix was based on adherence to the *Australian Guide to Healthy Eating* [37], combined with international sports nutrition recommendations [3], and is described elsewhere [36]. Briefly, a total ADI score (out of a possible 125) is calculated from the sum of the individual ADI components; where a higher score indicates greater compliance with dietary recommendations (i.e., total score ≥ 90 was classified as *exceeds recommendations*, a score from 66 to 89 was classified as *meets recommendations*, while a score ≤ 65 was classified as *below recommendations*). Specifically, the ADI evaluates the dietary intake of core foods (i.e., fruit, vegetables, grains, breads and cereals, dairy and alternatives, plus meat and alternatives), discretionary foods, and alcohol consumed over the past seven days (i.e., *Core Nutrition* sub-score). Servings of fruit, vegetables, grains, dairy, fish, eggs and plant-protein alternatives, discretionary foods, and alcohol were assessed using standard servings as outlined by the *Australian Guide to Healthy Eating* [37], while lean red meat and poultry were evaluated using typically consumed portions [39,40]. The ADI also assesses dietary indicators for specific micronutrients, such as calcium and iron, that might have increased requirements in athletes (i.e., *Special Nutrients* sub-score), and patterns of dietary behaviours specific to athletes undertaking a rigorous training schedule (i.e., *Dietary Habits* sub-score). Information about special diets or intolerances, culinary skills, current training load, and recent sports supplement use was also captured but not scored. Athlete responses to non-scored items were included in the feedback report provided to the ASDs to facilitate context to the self-reported dietary intake of the athletes. The total ADI score, sub-scores, and non-scored information were used in combination to provide an indication of overall diet quality and dietary patterns of the athletes. An example of an athlete’s ADI results, including the individual scores achieved for dietary components within each sub-category, sub-scores, and the total ADI score, is presented in Supplementary Material S1.

### 2.4. Evaluation Survey for Sports Dietitians

Following the completion of the athlete recruitment and participation phase, ASDs were asked to respond to a brief electronic survey to assess the perceived benefits of using the ADI in sports nutrition practice (Supplementary Material S2). The survey asked the ASDs to list the sporting program(s) where their athletes used the ADI, then asked them to rate their perceived agreement from 1 to 5 (i.e., 1 = *strongly agree* to 5 = *strongly disagree*) to twelve (*n* = 12) survey items. Survey items evaluated the perceived usefulness of the electronic ADI to support the nutrition care of athletes, and the perceived helpfulness of the ADI to identify athletes with poor diet quality, to prioritise nutrition support, and to advocate for sports nutrition services. Other survey items involved rating the perceived usefulness of the automatic scoring mechanism and the feedback report, in addition to rating the acceptability of using the electronic tool with athletes. An option to provide open-ended feedback about the ADI was also provided. The ASDs were sent two follow-up reminders via e-mail to complete the online survey, before receiving a third and final reminder to participate in the survey. The number of ASDs who did not complete the online survey was documented. The evaluation survey was developed using Qualtrics software (Qualtrics, 2019, Provo, UT, USA) and was administered via a web link housed on a university server.

### 2.5. Statistical Analysis 

Descriptive analyses are presented as the mean values and standard deviations for demographics, total ADI scores and sub-scores, and dietary intake data (i.e., servings of core foods, discretionary foods, and alcohol reported within the ADI). Data were checked for normality using Shapiro–Wilk tests and histograms for curves, skewness and kurtosis. A comparison of mean scores and dietary intake of core foods, discretionary foods, and alcohol based on sex was conducted using independent *t* tests. Differences between mean total scores and scores awarded for the specific dietary indicator components for calcium and iron (i.e., <8 and ≥8, out of a possible 10 points each; where a score of ≥8 was for a dietary intake that met the recommended servings of dairy and lean red meat, respectively) [37] were also assessed using independent *t* tests. Analysis of variance (ANOVA) and the Bonferroni multiple comparison methods were used to determine relationships between ADI scores and demographics plus sport-specific variables based on sex; where some categories were collapsed for reporting purposes (i.e., representative calibre, stage of training cycle, weekly training hours). Correlation between ADI scores and demographics was evaluated using Spearman’s rank correlation coefficient (r_s_). The strength of Spearman’s correlation was interpreted as low (0.30–0.50), moderate (0.50–0.70), high (0.70–0.90), or very high (≥0.90) [41]. All statistical analyses were performed using IBM SPSS Statistics version 26.0 (IBM Corp, Armonk, NY, USA) with the significance level accepted as *p* < 0.05.

## 3. Results

One hundred and sixty-eight elite athletes were invited to participate in this study between December 2019 and May 2020. Of the 168 athletes who initially consented, three (1.8%) athletes did not complete the ADI despite the follow-up reminders. Analyses were undertaken on the remaining 165 athletes. Demographic and sport-specific characteristics are presented in Table 1. Athlete participants represented team (i.e., basketball, cricket, hockey, netball, water polo), and individual (i.e., artistic swimming, cycling, equestrian, golf, rowing, tennis, track and field) sports. Most athletes were competing at an international (62.4%) level (Table 1).

The athlete responses to a selection of non-scored items are presented in Table 2. Iron deficiency was reported by over one-third of all athletes, while more than half of females reported they currently take a prescribed medication, such as the oral contraceptive pill (OCP). More than one-quarter of athletes reported that they *always* or *usually* follow one or more special diets, such as dairy or lactose-free (*n* = 25), food allergy or intolerance (*n* = 11), or a vegetarian diet (*n* = 10). More than three-quarters of all athletes reported using sports and dietary supplements over the past four weeks (Table 2).

### 3.1. Diet Quality of Athletes

Athletes completed the ADI in 20.4 ± 7.4 min (median 19.3 min; range: 8.6 to 58.4 min). The mean total ADI score for all athletes was 91.4 ± 12.2 (range 53 to 117, out of a possible 125). Over half (56%) of athletes (*n* = 93) had a total score ≥ 90 (i.e., *exceeds recommendations*), and 42% (*n* = 70) had a total score from 66 to 89 (i.e., *meets recommendations*), while 1% of athletes (*n* = 2) scored ≤ 65 (i.e., *below recommendations*). Further examination of the responses by two athletes who had low ADI scores indicated a low intake of core foods (i.e., vegetables, grains, dairy products, and/or lean red meat), and low scores in the specific dietary indicator components for iron and calcium. Mean sub-scores for *Core Nutrition*, *Special Nutrients*, and *Dietary Habits* for all athletes (*n* = 165) were 57.6 ± 9.3 (range 33 to 76, out of a possible 80), 25.1 ± 4.1 (range 10 to 35, out of a possible 35), and 8.6 ± 1.6 (range 2 to 10, out of a possible 10), respectively (Table 3).

Differences in mean total scores were compared between the athletes who scored < 8 and ≥8 points (out of a possible 10) in the specific dietary indicator components for calcium and iron. Mean total scores were higher for those athletes who scored ≥ 8 in the dietary indicator components for calcium (95.1 vs. 81.3; *p* < 0.0001), and iron (92.8 vs. 88.4; *p* < 0.05) compared to those athletes who scored < 8 in the dietary indicator components for calcium and iron, respectively.

Overall, there was no association between mean total scores and age (*p* = 0.22), self-reported body mass (*p* = 0.24), or BMI (*p* = 0.86) for all athletes (*n* = 165). There were no differences in mean total scores according to demographic and sports-specific variables, with the exception of athletes involved in team sports who had a higher mean total score compared to individual sport athletes (92.7 vs. 88.5, *p* < 0.05) (Table 4). Between-group differences were also observed in *Dietary Habits* sub-scores of athletes training fewer hours (i.e., 0–11 h/week) who scored higher compared to athletes training ≥ 12 h/week (*p* < 0.05) (Table 4).

For males, those currently in the off season had a higher mean total score than those currently in the in season (*p* = 0.04). Additionally, *Special Nutrients* sub-scores were higher for males in the off season (vs. pre-season; *p* = 0.02; and vs. in season; *p* = 0.002), and *Dietary Habits* sub-scores were higher for male team sport athletes (vs. individual sport athletes; *p* < 0.05). For females, *Dietary Habits* sub-scores were higher in those training fewer hours per week (vs. training ≥ 12 h/week; *p* = 0.02), those currently in the pre-season (vs. off season, *p* = 0.04), and those who had basic cooking skills (vs. advanced cooking skills; *p* < 0.001). No other between-group differences were observed in ADI scores based on sex.

Self-reported dietary intake data (i.e., servings of core foods, discretionary foods, and alcohol), derived from the *Core Nutrition* sub-category, is presented in Table 5. There were no differences in mean servings of fruit, vegetables, discretionary foods, or alcohol based on sex; however, female athletes reported a lower mean intake of grains (3.9 vs. 5.4 servings; *p* < 0.01), dairy (2.5 vs. 3.0; *p* < 0.01), and meat and alternatives (1.7 vs. 2.2; *p <* 0.001) compared to male athletes, respectively (Table 5).

### 3.2. Sports Dietitians’ Evaluation Survey

Sixteen (*n* = 16) ASDs recruited one or more athletes who consented to participate in this study. Of the sixteen ASDs, four (25%) did not respond to the evaluation survey despite the follow-up reminders. Responding ASDs (*n* = 12) rated their perceived agreement as *somewhat agree* to *strongly agree* for nine (75%) of the twelve survey items (Supplementary Material S3). Two ASDs or fewer rated the remaining three survey items as *neither agree nor disagree* or *somewhat disagree* (Supplementary Material S3).

Most (75%) ASDs *strongly agreed* to the items on the perceived usefulness of the ADI to support the nutrition care of athletes; the helpfulness of the ADI for screening athletes and identifying areas for nutrition support; the helpfulness of the automatic scoring mechanism and feedback report; and to advocate for sports nutrition services (Supplementary Material S3). The acceptability of the ADI to support the nutrition care of athletes and feasibility for its use was also rated as *strongly agree* by more than 75% of ASDs surveyed. Specific comments concerning the feedback report were identified and incorporated into a revised version of the automated report. 

## 4. Discussion

This is the first study to investigate the diet quality of elite Australian athletes using the recently validated electronic ADI. The majority of athletes had a high total score, as indicated by the median score of 91.0 (range 53 to 117). No association between the total ADI score and demographic variables such as age [22,24], education level [28], and representative calibre [28] were found, as previously reported in other diet quality studies in athletes. Team sport athletes scored higher than individual sport athletes (92.7 vs. 88.5, *p* < 0.05). Between-group differences were also observed in *Dietary Habits* sub-scores of athletes training fewer hours (i.e., ≤11 h/week) who scored higher compared to athletes training longer (i.e., ≥12 h/week; *p* < 0.05). The difference observed in mean scores based on primary sport may have been due to the range of different types of team sports, in addition to the individual types of sports (i.e., aesthetic, skill, and endurance sports), included in this study. Sub-scores and non-scored items provided important contextual elements for interpretation of the overall results. Evaluation data indicated that the ADI is suitable for use in assessment of diet quality in practice. 

While the overall diet quality of the athletes was comparable to the findings of other studies in athletes [2,12,23], and was higher than the diet quality reported in young, Australian adults [42], heterogeneity in methodology and scoring algorithms make comparison of absolute scores between diet quality studies difficult. Specifically, the scoring applied in population indices typically involves a comparison to county-specific dietary guidelines, whereas the ADI score is derived from a combination of three sub-scores (i.e., *Core Nutrition*, *Special Nutrients*, and *Dietary Habits*) and is based on adherence to dietary guidelines [37] combined with sports nutrition recommendations [3]. However, those studies that have compared diet quality scores between athlete and non-athlete participants suggest that athletes achieve higher scores [2,23,27]. Athletes’ access to professional dietary guidance and nutrition education, along with the emphasis on the role of optimal nutrition for performance, may improve the diet quality of athletes [2,43]. However, it is important that the total ADI score is examined in context with other indicators assessed by the ADI (such as the dietary indicators for special nutrients and dietary habits), in addition to non-scored items that are reported in the ADI.

Evaluation of the self-reported dietary intake data (i.e., intake of core foods, discretionary foods, and alcohol) derived from the *Core Nutrition* sub-category indicated that some of the athletes did not achieve the recommended servings for some core foods (i.e., vegetables, grains, breads and cereals, plus meat and alternatives) as outlined in the *Australian Guide to Healthy Eating* [37]. Our findings are similar to others who have previously reported a sub-optimal intake of vegetables [11,12,13,26,27], and grains, breads and cereals [12,13] in athletes. In particular, an inadequate intake of grains, breads and cereals has been shown to reflect a carbohydrate intake below sports nutrition recommendations [12,13,35], supporting the need for further investigation to determine the adequacy and timing of carbohydrate intake based on individual sporting requirements. Additionally, a sub-optimal intake of grains, breads and cereals (4.4 servings), as well as meat and alternatives (1.9 servings) in the current study is comparable to the findings of the 2017–2018 *National Health Survey for Australians* (4.7 and 2.0 servings, respectively) [44]. However, it was possible that the ADI under-estimated the total number of meat and alternatives servings, due to the difference in quantification of serving size specifically involving lean red meat and poultry in the ADI (i.e., typically consumed portion ~120 g) [39,40], as compared to the standard serving size (i.e., 65 g) of meat and alternatives outlined in the *Australian Guide to Healthy Eating* [37], and also referred to in the 2017–2018 *National Health Survey for Australians* [44]. Overall, the athletes had a higher intake of fruit (2.4 vs. 1.4), vegetable (3.6 vs. 2.9), plus dairy and alternative (2.7 vs. 1.5) servings compared to the general population of Australian adults [44]. In addition, the athletes consumed fewer servings of discretionary foods and alcohol than previously reported in Australian adults [44,45]. Therefore, our results suggest that elite Australian athletes have at least comparable, and likely better diets overall, when compared to young Australian adults [42,44,45].

Importantly, a dietary intake that includes all core food groups is essential for optimal health and is indicative of overall nutrient adequacy [35,37]. In particular, we previously reported a low to moderate correlation between mean ADI scores and the intake of iron (r_s_ = 0.44) and calcium (r_s_ = 0.59), respectively [35]. Additional analyses have indicated that athletes who scored ≥ 8 points in the dietary indicator components for dairy foods and lean red meat had higher calcium (1185.2 mg vs. 835.4 mg; *p* < 0.05) and iron (15.1 mg vs. 10.9 mg; *p* < 0.0001) intakes compared to athletes who scored < 8 points, respectively (unpublished data). In the present study, we found that mean total ADI scores were higher for those athletes who scored ≥ 8 (out of a possible 10) in the dietary indicator components for calcium and iron. An evaluation of these specific micronutrients (i.e., calcium and iron) is one of the unique features of the ADI compared to population indices. The food-based scoring methodology in the ADI indicates a range in athletes’ adherence to dietary recommendations, and may therefore assist ASDs in tailoring advice on specific areas of dietary concern. Further investigation of the association between ADI scores and measures of iron status or indices for bone health would be valuable.

Examination of the ADI sub-scores indicates habits associated with those athletes who train for longer (i.e., ≥12 h/week) compared to athletes who train fewer hours per week (i.e., ≤11 h/week). Potential factors that may influence the dietary habits of athletes who train for longer (i.e., a rigorous training schedule that may involve one or more training sessions a day, conducted over 5 or more days a week) include the ability to appropriately plan meals and organise nutrition to support training [14,46]. Furthermore, a possible delay in nutritional intake due to gastrointestinal discomfort or suppressed appetite following exercise [5,14], and general time-management skills of athletes with possible co-commitments such as work, study, or travel, may also influence athletes’ dietary intake. Other factors that may influence the dietary intake of athletes include nutrition knowledge, dietary preferences or intolerances, and food management skills such as purchasing and preparing food [46]. While some of these are reported in the ADI, an evaluation of dietary intake assessed by the ADI compared to sports nutrition knowledge assessed by the PEAKS-NQ would be worthwhile. Furthermore, differences observed in the sub-scores associated with the stage of the training cycle suggest the importance of measuring diet quality longitudinally across a competitive season (i.e., pre-season vs. in season). As the electronic ADI evaluates dietary intake based on the previous seven days, fluctuations in dietary intake due to the periodisation of training over the micro- (i.e., 1 week), meso- (i.e., 2 to 6 weeks), and macro-cycle (i.e., competitive season) [3,6,7,8,9,10,11,12,13,14,15,16,17,18,19,20,21,22,23,24,25,26,27,28,29,30,31,32,33,34,35,36,37,38,39,40,41,42,43,44,45,46,47,48,49] support the importance of longitudinal measurement.

Lastly, the ASDs’ responses to the evaluation survey support the overall usefulness of the ADI in the nutrition care of athletes, and the utility of the ADI scoring and feedback report for ASDs in practice. While the self-administered electronic ADI does not replace a more detailed dietary assessment of athletes, it can be completed independently of ASDs’ involvement and in less time (median 19.3 min) compared to other dietary assessment methods such as face-to-face in-depth dietary interviews, assisting ASDs to efficiently screen or monitor athletes involved in high-performance sporting programs, identify key areas for nutrition support, and help advocate for sports nutrition services. Specifically, the special dietary needs reported by the athletes (i.e., 25% reporting food allergy or intolerance), and the high prevalence of sports supplement use (i.e., 81% reporting using sports and dietary supplements in the past 4 weeks) further emphasize the need for ASDs in providing evidence-based nutrition interventions to athletes.

### Strengths and Limitations

One of the strengths of this study is that the elite athletes were of a higher calibre than athletes in previous diet quality studies [12,22,26,27]. In addition, our sample size was greater than other diet quality studies in athletes (range 18 to 138 participants) [2,12,22,24,26,27,28]. While there were a high proportion of female athletes in our study, no differences were observed in mean ADI scores based on sex. However, further research with a larger representative sample, involving athletes with differing physiological requirements (i.e., skill, endurance, power/strength, and weight class sports), would be beneficial. Despite technological advances in dietary assessment methodology, self-reported dietary intake is still prone to bias in accuracy of dietary recall [5,6], portion estimation [5,50], or mis- or under-reporting of dietary intake [5,6,51]. Further evaluation of the ADI compared to nutritional biomarkers may assist in providing information about and improving the accuracy of the self-reported dietary assessment [52]. Finally, the high ADI scores may have been due to the elite athletes having had prior access to sport nutrition services due to their involvement in high-performance sporting programs. However, limited resourcing could restrict interaction of some athletes with ASDs [2,7]. Therefore, a comparison between those athletes who receive nutrition support compared to those who do not have access to an ASD, in addition to administering the tool in a cohort of lower-calibre or developing athletes is needed to demonstrate score discrimination based on these factors.

## 5. Conclusions

This is the first study that evaluates diet quality of elite Australian athletes using the recently validated ADI. This study demonstrates that athletes had high overall ADI scores and that the self-administered dietary assessment tool can provide a useful way to rapidly evaluate usual dietary intake and dietary habits of athletes. While the athletes reported a sub-optimal intake of some core foods compared to population recommendations, overall, their dietary intake was at least comparable to, or likely better than, the general population of Australian adults. The usefulness of the ADI to assist in the rapid screening of athletes, direct ASDs’ attention to specific areas of dietary concern, and tailor nutrition intervention strategies was confirmed by the evaluation survey in ASDs. Further investigation of the diet quality of athletes measured longitudinally over a competitive season and in lower-calibre athletes, plus validation of ADI scores compared to measures of iron status or indices of bone health, is warranted.

## Figures and Tables

**Table 1:** Athlete demographic and sports-specific characteristics.

Participant Characteristics	Total (*n* = 165)		Female (*n* = 112)		Male (*n* = 53)	
	Mean ^a^	SD ^a^	Mean ^a^	SD ^a^	Mean ^a^	SD ^a^
Age (years)	20.0 ^a^	(5.0) ^a^	20.0 ^a^	(4.0) ^a^	21.0 ^a^	(4.0) ^a^
Athletes 16–19 y **N** (%)	71 (43.0)		53 (47.3)		18 (34.0)	
Athletes > 20 y **N** (%)	94 (57.0)		59 (52.7)		35 (66.0)	
Body mass ^^^ (kg)	73.6	12.0	70.9 ***	11.1	79.2 ***	12.1
Stature ^^^ (cm)	178.0 ^a^	(13.5) ^a^	175.0 ^a^***	(13.0) ^a^	182.0 ***	10.7
BMI (kg·m^2^)	23.1	2.4	22.8 *	2.4	23.8 *	2.1
*Highest education level***N** (%)						
Secondary school ^†^	121 (73.3)		82 (73.2)		39 (73.6)	
University/TAFE	44 (26.7)		30 (26.8)		14 (26.4)	
*Primary sport***N** (%)						
Team sports ^1^	113 (68.5)		80 (71.4)		33 (62.3)	
Individual sports ^2^	52 (31.5)		32 (28.6)		20 (37.7)	
*Representative calibre***N** (%)						
State	5 (3.0)		5 (4.5)		0 (0)	
National	57 (34.5)		39 (34.8)		18 (34.0)	
International **N** (%)	103 (62.4)		68 (60.7)		35 (66.0)	
*Junior international*	15 (9.1)		10 (8.9)		5 (9.4)	
*Age international*	38 (23.0)		23 (20.5)		15 (28.3)	
*Open international*	50 (30.3)		35 (31.2)		15 (28.3)	
*Stage of training cycle***N** (%)						
Pre-season	75 (45.4)		51 (45.5)		24 (45.3)	
In season	27 (16.4)		17 (15.2)		10 (18.9)	
Off season	51 (30.9)		38 (33.9)		13 (24.5)	
Injured/rehabilitation	12 (7.3)		6 (5.4)		6 (11.3)	
*Weekly training hours***N** (%)						
0–4 h/week	3 (1.8)		1 (0.9)		2 (3.8)	
5–11 h/week	41 (24.8)		26 (23.2)		15 (28.3)	
12–15 h/week	58 (35.2)		45 (40.2)		13 (24.5)	
16–20 h/week	45 (27.3)		30 (26.8)		15 (28.3)	
20+ h/week	18 (10.9)		10 (8.9)		8 (15.1)	

ADI, Athlete Diet Index; SD, standard deviation; y, years; BMI, body mass index; TAFE, Technical and Further Education (an Australian tertiary education system offering courses mainly in technical and vocational subjects); ^a^ median and interquartile range (IQR) values reported for non-normally distributed demographic data; ^^^ self-reported body mass and stature; ^†^ secondary school students usually turn 18 years of age in their final year of secondary school in Australia; ^1^ team sports included basketball, cricket, hockey, netball, and water polo; ^2^ individual sports included artistic swimming, cycling, equestrian, golf, rowing, tennis, and track and field; *p* value applies to the difference in demographics between female and male athletes, * *p* < 0.05; *** *p* < 0.001.

**Table 2:** Overview of non-scored ADI responses including medical and dietary information, sport and dietary supplement use, plus culinary habits.

Responses to Items Regarding Medical and Dietary Information		N (%)
*Currently taking prescribed medication* (specified type)		61 (37.0)
	OCP	36 (21.8)
	Iron, calcium, vitamin D	7 (4.2)
	Antibiotics	3 (1.8)
	Asthma treatment	3 (1.8)
	NSAIDs	2 (1.2)
	Anti-depressants	2 (1.2)
	Acne treatment	2 (1.2)
	Other medications/NS	11 (6.7)
*Previously diagnosed with a nutritional deficiency*		62 (37.6)
(specified type)	Iron	60 (36.4)
	Vitamin B12	1 (0.6)
	Vitamin D	1 (0.6)
*Regularly follow a special diet(s) ^#^* (specified type)		42 (25.4)
	Low lactose/dairy free	25 (15.2)
	Food allergy/intolerance	11 (6.7)
	Vegetarian/vegan	10 (6.1)
	Gluten free	8 (4.8)
	Paleo/high protein	6 (3.6)
	Intermittent fasting	5 (3.0)
	Ketogenic/high fat	3 (1.8)
	Other type of diet/NS	5 (3.0)
*Supplement use in past 4 weeks* (specified type)		134 (81.2)
	Sports foods ^1^	125 (75.8)
	Dietary supplements ^2^	81 (49.1)
	Sports supplements ^3^	31 (18.8)
**Responses to items regarding culinary habits**		
*How often you are the main person who grocery shops?*	Rarely/Never	47 (28.5)
	Sometimes	51 (30.9)
	Always/Usually	67 (40.6)
*How often you are the person who prepares main meals?*	Rarely/Never	57 (34.5)
	Sometimes	45 (27.3)
	Always/Usually	63 (38.2)
*Rate your perceived cooking ability*	Basic cook/I don’t cook	36 (21.8)
	Intermediate cook	93 (56.4)
	Advanced cook	36 (21.8)

ADI, Athlete Diet Index; OCP, oral contraceptive pill; NSAIDs, non-steroidal anti-inflammatory drugs; NS, not specified; ^#^ participant responded that they *always* or *usually* follow one or more special diet; ^1^ sports foods included sports drink, sports bars, carbohydrate gels, protein powders; ^2^ dietary supplements included multi-vitamins, omega 3 fatty acids, iron supplements, probiotics; ^3^ sports supplements included creatine, beta-alanine, bicarbonate, and caffeine.

**Table 3:** Mean total ADI scores and sub-scores for all athletes (*n* = 165), and the difference in mean scores between female and male athletes.

	Total		Female		Male		Mean Difference	
	Mean	SD	Mean	SD	Mean	SD	(95% CI)	*p* Value ^‡^
Total ADI score ^#^	91.4	12.2	91.0	12.1	92.1	12.7	−1.0 (−5.0, 3.0)	0.62
*Core Nutrition* sub-score ^#^	57.6	9.3	57.5	9.1	57.9	10.0	−0.4 (−3.5, 2.7)	0.80
*Special Nutrients* sub-score ^#^	25.1	4.1	24.9	4.1	25.6	4.1	−0.6 (−1.9, 0.7)	0.36
*Dietary Habits* sub-score ^#^	8.6	1.6	8.6	1.5	8.6	1.8	0.0 (−0.5, 0.6)	0.94

ADI, Athlete Diet Index; SD, standard deviation; ^#^ the total ADI score out of a possible 125, *Core Nutrition* sub-score out of a possible 80, *Special Nutrients* sub-score out of a possible 35, *Dietary Habits* sub-score out of a possible 10; ^‡^ *p* value applies to the difference in mean ADI total score and sub-scores between female (*n* = 112) and male (*n* = 53) athletes.

**Table 4:** Mean total ADI scores and sub-scores for all athletes (*n* = 165) and the difference in mean total ADI scores between groups based on demographics and sports-specific characteristics.

Demographic Variables	*Core Nutrition* Sub-Score ^#^		*Special Nutrients* Sub-Score ^#^		*Dietary Habits* Sub-Score ^#^		Total ADI Score ^#^		Mean Difference ^¶^	
	Mean	SD	Mean	SD	Mean	SD	Mean	SD	(95% CI)	*p* Value ^‡^
*Athletes*										
Female (*n* = 112)	57.5	9.1	24.9	4.1	8.6	1.5	91.0	12.1	−1.0 (−5.0, 3.0)	0.62
Male (*n* = 53)	57.9	10.0	25.6	4.1	8.6	1.8	92.1	12.7		
*Age*									1.7 (−2.1, 5.5)	0.39
16–19 y (*n* = 71)	58.4	10.2	25.1	4.1	8.8	1.3	92.3	13.3		
>20 y (*n* = 94)	57.0	8.6	25.2	4.2	8.5	1.8	90.6	11.4		
*Highest level of education*									1.3 (−2.9, 5.6)	0.55
Secondary (*n* = 121)	57.2	8.9	25.1	4.1	8.7	1.6	91.7	12.3		
Tertiary (*n* = 44)	57.8	9.6	25.3	4.3	8.4	1.5	90.4	12.1		
*Main grocery shopper*									*F* (*df*) 0.001 (2162)	0.99
Never/Rarely (*n* = 47)	57.5	10.0	25.0	4.5	8.8	1.7	91.3	13.5		
Sometimes (*n* = 51)	57.6	10.0	25.2	3.8	8.6	1.5	91.4	12.7		
Usually/Always (*n* = 67)	57.7	8.4	25.2	4.1	8.5	1.6	91.4	11.1		
*Main person who prepares meals*									*F* (*df*) 1.149 (2162)	0.32
Never/Rarely (*n* = 57)	55.2	9.9	25.4	3.5	8.7	1.7	89.4	12.7		
Sometimes (*n* = 45)	58.6	8.4	25.2	4.2	8.7	1.4	92.5	11.5		
Usually/Always (*n* = 63)	59.0	9.1	24.8	4.6	8.5	1.6	92.3	12.3		
*Self-rated cooking ability*									*F* (*df*) 0.126 (2162)	0.88
Basic/Don’t cook (*n* = 36)	57.1	9.1	24.9	3.9	9.1	1.5	91.1	11.4		
Intermediate (*n* = 93)	57.2	9.2	25.3	4.3	8.6	1.6	91.1	12.4		
Advanced (*n* = 36)	59.2	9.9	24.9	4.0	8.2	1.6	92.3	13.0		
*Sport-specific variables*										
Primary sport ^^^									−4.2 (−8.3, −0.2)	0.04 *
Individual (*n* = 52)	55.7	9.5	24.3	4.4	8.5	1.8	88.5	13.2		
Team (*n* = 113)	58.4	9.1	25.5	3.9	8.7	1.5	92.7	11.6		
*Representative calibre*									−0.9 (−4.8, 3.0)	0.65
State/National (*n* = 62)	57.2	8.9	25.0	3.6	8.7	1.6	90.8	10.8		
International (*n* = 103)	57.8	9.6	25.2	4.4	8.6	1.6	91.7	13.1		
*Stage of training cycle*									*F* (*df*) 1.904 (2162)	0.15
Pre-season (*n* = 75)	59.1	9.1	24.8	4.1	8.8	1.5	92.7	11.7		
In season (*n* = 27)	54.6	10.4	24.3	4.2	8.5	1.6	87.4	13.7		
Off season, injured (*n* = 63)	57.0	8.9	25.9	4.0	8.5	1.7	91.4	12.0		
*Weekly training hours*									*F* (*df*) 0.004 (2162)	0.99
0–11 h/week (*n* = 44)	56.9	9.8	25.4	4.2	9.2 ^a^	1.6	91.5	13.0		
12–15 h/week (*n* = 57)	57.8	9.3	24.7	4.3	8.8 ^b^	1.4	91.4	12.4		
16+ h/week (*n* = 64)	57.9	9.2	25.4	3.9	8.0 ^a,b^	1.8	91.3	11.7		

ADI, Athlete Diet Index; h, hours; ^#^ the total ADI score out of a possible 125, *Core Nutrition* sub-score out of a possible 80, *Special Nutrients* sub-score out of a possible 35, *Dietary Habits* sub-score out of a possible 10; ^¶^ differences for variables involving more than two groups were assessed by analysis of variance (ANOVA) and values are listed as *F*, F value (i.e., mean between-group variance divided by the mean within-group variance) and *df*, degrees of freedom; ^^^ primary sports were classified as individual (i.e., artistic swimming, cycling, equestrian, golf, rowing, tennis, track and field), and team (i.e., basketball, cricket, hockey, netball, water polo) sports; ^‡^ *p* value applies to the mean between group difference in mean total ADI scores; * *p* < 0.05, while ^a^ *p* < 0.001 applies to the post-hoc Bonferroni pairwise mean between-group difference observed in mean ADI sub-scores between groups of athletes training 0–11 and ≥16 h/week, respectively; and ^b^ *p* < 0.05 applies to the post-hoc Bonferroni pairwise mean between-group difference observed in mean ADI sub-scores between groups of athletes training 12–15 and ≥16 h/week, respectively.

**Table 5:** Mean servings of core foods, discretionary foods, and alcohol reported by all athletes (*n* = 165), and the difference in mean servings between female and male athletes.

Dietary Intake	Total		Female		Male		Mean Difference	
(Servings/day)	Mean	SD	Mean	SD	Mean	SD	(95% CI)	*p* Value ^‡^
Fruit	2.4	1.0	2.4	0.9	2.4	1.1	0.4 (−0.3, 0.4)	0.81
Vegetables	3.6	1.1	3.7	1.1	3.5	1.3	0.2 (−0.2, 0.6)	0.38
Grain, breads, cereals	4.4	3.0	3.9	2.5	5.4	3.7	−1.5 (−2.6, 0.4)	<0.01
Dairy and alternatives	2.7	1.3	2.5	1.2	3.0	1.3	−0.6 (−0.9, −0.1)	<0.01
Meat and alternatives ^#^	1.9	0.6	1.7	0.5	2.2	0.8	−0.4 (−0.6, −0.3)	<0.001
Discretionary foods ^#^	1.1	0.7	1.0	0.6	1.1	0.8	−0.1 (−0.3, 0.2)	0.62
Alcohol ^^^	1.7	0.6	1.8	0.5	1.6	0.7	0.2 (0.0, 0.4)	0.07

ADI, Athlete Diet Index; SD, standard deviation; ^#^ combined reported weekly servings divided by 7; ^^^ standard servings of alcohol per week; ^‡^ *p* value applies to the mean of the difference in servings of core foods, discretionary foods and alcohol between female (*n* = 112) and male (*n* = 53) athletes

## Data Availability

Data Availability Statement: The data that support the findings of this study is available from the University of Sydney, but restricted for research use only. The data are not publicly available. Data are available from the authors upon reasonable request and with permission of the University of Sydney.

## Supplementary Materials

The following are available online at https://www.mdpi.com/2072-6643/13/1/126/s1, Supplementary Material S1: Exemplar of an athlete participant’s ADI results including the individual scores achieved for the dietary components within each sub-category, sub-scores and the total ADI score; Supplementary Material S2: Evaluation survey items for the sports dietitians and developed on Qualtrics^TM^; Supplementary Material S3: Rating of level of agreement to survey items by the accredited sports dietitians (ASDs) who completed the electronic evaluation survey.
