# Supplementary material for: Diet Quality of Elite Australian Athletes Evaluated Using the Athlete Diet Index
Source: Nutrients. 2020 Dec 31;13(1):126. doi: 10.3390/nu13010126 (PMC7823332; doi:10.3390/nu13010126)
Supplement: Supplementary file 1 [file nutrients-13-00126-s001.pdf]

Supplementary Material S1. Exemplar of an athlete's ADI results including the scores achieved for the individual dietary components within each sub-category, sub-scores, and total ADI score

Core Nutrition	Maximum	Your score
Intake of fruit	10	10
Variety of types of fruit	5	5
Intake of vegetables	10	8
Variety of types of vegetables	5	4
Intake of grains, breads, and cereals	10	5
Consume wholegrain bread more often	5	0
Intake of dairy and dairy alternatives	10	8
Consume reduced fat milk more often	5	3
Intake of lean meat and protein alternatives	10	2
Intake of discretionary foods:		
Sweet and savoury snack foods	3	2
Processed meats	1	1
Frequency of eating out of home/takeaway food	3	3
Intake of alcohol	3	1
**sub-score**	80	52

Special Nutrients - Dietary Indicators	Maximum	Your score
Foods rich in essential fatty acids	5	3
Foods rich in antioxidant vitamins	10	5
Foods rich in the specific micronutrients:		
Calcium	10	8
Iron	10	5
**sub-score**	35	21

Dietary Habits	Maximum	Your score
Healthy dietary pattern	2	2
Distribution of food intake over the day	2	1
Nutrition practices that support training:		
Eating before training	2	2
Eating after training	2	1
Hydration choices	2	2
**sub-score**	10	8
**Total ADI score** (H > 90; M 66-89; L < 65)	**125**	**81**

ADI, Athlete Diet Index; H, high; M, medium; L, low

Supplementary Material S2. Evaluation survey items for the sports dietitians and developed on Qualtrics™

Please list the sports for athletes where you provide dietetic support and who used the dietary assessment tools

____

____

____

Please select the most appropriate response for each tool (i.e. PEAKS-NQ & ADI)

Q1. The tools helped to provide useful information which I could use to support the nutrition care of my athletes

	Strongly agree	Somewhat agree	Neither agree nor disagree	Somewhat disagree	Strongly disagree
PEAKS-NQ	○	○	○	○	○
ADI	○	○	○	○	○

Q2. The tools helped with prioritising nutrition support to specific athletes

	Strongly agree	Somewhat agree	Neither agree nor disagree	Somewhat disagree	Strongly disagree
PEAKS-NQ	○	○	○	○	○
ADI	○	○	○	○	○

Q3. The tools helped me to screen the athletes and determine what kind of nutrition support

they needed most

	Strongly agree	Somewhat agree	Neither agree nor disagree	Somewhat disagree	Strongly disagree
PEAKS-NQ	○	○	○	○	○
ADI	○	○	○	○	○

Q4. The tools helped me to identify athletes with low levels of nutrition knowledge or those that needed to improve their diets that might have not been identified otherwise

	Strongly agree	Somewhat agree	Neither agree nor disagree	Somewhat disagree	Strongly disagree
PEAKS-NQ	○	○	○	○	○
ADI	○	○	○	○	○

Q5. The tools provided information on knowledge and use of dietary supplements and other eating behaviours and practices that cause concern in athletes under my care

	Strongly agree	Somewhat agree	Neither agree nor disagree	Somewhat disagree	Strongly disagree
PEAKS-NQ	○	○	○	○	○
ADI	○	○	○	○	○

Q6. I would use these tools again to support the nutrition care of athletes

	Strongly agree	Somewhat agree	Neither agree nor disagree	Somewhat disagree	Strongly disagree
PEAKS-NQ	○	○	○	○	○
ADI	○	○	○	○	○

Q7. It was useful to have total and section scores.

	Strongly agree	Somewhat agree	Neither agree nor disagree	Somewhat disagree	Strongly disagree
PEAKS-NQ	○	○	○	○	○
ADI	○	○	○	○	○

Q8. It was practical and time efficient for the athletes to complete the tools electronically

	Strongly agree	Somewhat agree	Neither agree nor disagree	Somewhat disagree	Strongly disagree
PEAKS-NQ	○	○	○	○	○
ADI	○	○	○	○	○

Q9. It was helpful to have automated scoring and feedback after the athletes completed each tool

	Strongly agree	Somewhat agree	Neither agree nor disagree	Somewhat disagree	Strongly disagree
PEAKS-NQ	○	○	○	○	○
ADI	○	○	○	○	○

Q10. I would use these tools to justify and advocate to the sport/coach and administration to continue or enhance the nutrition servicing I provide to the athletes under my care

	Strongly agree	Somewhat agree	Neither agree nor disagree	Somewhat disagree	Strongly disagree
PEAKS-NQ	○	○	○	○	○
ADI	○	○	○	○	○

Questions 11-12 are asking you about the usefulness of the feedback provided

Q11. The feedback report was informative, useful and athlete friendly

	Strongly agree	Somewhat agree	Neither agree nor disagree	Somewhat disagree	Strongly disagree
PEAKS-NQ	○	○	○	○	○
ADI	○	○	○	○	○

Q12. The feedback report provided a useful way for me to follow-up with the athlete and address specific issues to support their nutrition care

	Strongly agree	Somewhat agree	Neither agree nor disagree	Somewhat disagree	Strongly disagree
PEAKS-NQ	○	○	○	○	○
ADI	○	○	○	○	○

Any other comments/feedback:

____

____

Supplementary Material S3. Rating of level of agreement to survey items by the accredited sports dietitians (ASDs) who completed the electronic evaluation survey

**Survey item**	**Strongly agree (%)**	**Somewhat agree (%)**	**Neither agree nor disagree (%)**	**Somewhat disagree (%)**	**Strongly disagree [illegible]**
**Usefulness of the ADI**					
1. Provided useful information to support the nutrition care of my athletes	9 (75)	3 (25)			
2. Provided information on eating practices of concern and supplement use	5 (42)	5 (42)	1 (8)	1 (8)	
**Enhancement of sports nutrition practice**					
3. Helpful for screening and identifying key areas for nutrition support	10 (83)	2 (17)			
4. Helpful for identifying athletes with poor diet quality	7 (58)	5 (42)			
5. Helped prioritise nutrition support for specific athletes	6 (50)	6 (50)			
6. Would use to justify and advocate for nutrition services to athletes	10 (83)	2 (17)			
**Automated feedback report**					
7. Useful to receive the overall ADI and section scores	7 (58)	5 (42)			
8. Automated scoring and the feedback report were helpful	12 (100)				
9. Feedback report was informative, useful and athlete-friendly	7 (58)	4 (33)	1 (8)		
10. Provided a useful way to follow-up and address specific issues with athletes	8 (67)	4 (33)			
**Feasibility of the ADI**					
11. Practical and time efficient for athletes to complete electronically	10 (83)	1 (8)	1 (8)		
**Acceptability of the ADI**					
12. Would use the tool again to support the nutrition care of athletes	9 (75)	3 (25)			

ADI, Athlete Diet Index; ASDs, Accredited Sports Dietitians (n=12)
